# Microbiome analysis of thai traditional fermented soybeans reveals short-chain fatty acid-associated bacterial taxa

**DOI:** 10.1038/s41598-023-34818-0

**Published:** 2023-05-10

**Authors:** Thidathip Wongsurawat, Sawannee Sutheeworapong, Piroon Jenjaroenpun, Suvimol Charoensiddhi, Ahmad Nuruddin Khoiri, Supachai Topanurak, Chantira Sutthikornchai, Pornrutsami Jintaridth

**Affiliations:** 1 Division of Medical Bioinformatics, Research Department, Faculty of Medicine Siriraj Hospital, Mahidol University, Bangkok, 10700 Thailand; 2 Siriraj Long-Read Lab (Si-LoL), Faculty of Medicine Siriraj Hospital, Bangkok, 10700 Thailand; 3 Systems Biology and Bioinformatics Laboratory, Pilot Plant Development and Training Institute, King Mongkut’s University of Technology Thonburi, Bangkok, 10150 Thailand; 4 Department of Food Science and Technology, Faculty of Agro-Industry, Kasetsart University, Bangkok, 10900 Thailand; 5 Bioinformatics and Systems Biology Program, School of Bioresources and Technology and School of Information Technology, King Mongkut’s University of Technology Thonburi, Bangkok, 10150 Thailand; 6 Department of Molecular Tropical Medicine and Genetics, Faculty of Tropical Medicine, Mahidol University, Bangkok, 10400 Thailand; 7 Department of Protozoology, Faculty of Tropical Medicine, Mahidol University, Bangkok, 10400 Thailand; 8 Department of Tropical Nutrition and Food Science, Faculty of Tropical Medicine, Mahidol University, Bangkok, 10400 Thailand

**Keywords:** Biochemistry, Computational biology and bioinformatics, Microbiology

## Abstract

Thua Nao is a Thai traditional fermented soybean food and low-cost protein supplement. This study aimed to evaluate the bacterial community in Thua Nao from northern Thailand and assess potentially active short-chain fatty acids (SCFAs)-related bacteria. Sixty-five Thua Nao consisting of 30 wet and 35 dried samples were collected from six provinces: Chiang Rai, Chiang Mai, Mae Hong Son, Lampang, Lamphun, and Phayao. Bacterial diversity was significantly higher in the wet samples than in the dried samples. The dominant phyla were Firmicutes (92.7%), Proteobacteria (6.7%), Actinobacteriota (0.42%), and Bacteroidota (0.26%). The genus *Bacillus* (67%) was the most represented in all samples. *Lactobacillus*, *Enterococcus,* and *Globicatella* were enriched in the wet samples. Assessment of the SCFA-microbiota relationships revealed that high butyrate and propionate concentrations were associated with an increased *Clostridiales* abundance, and high acetate concentrations were associated with an increased *Weissella* abundance. Wet products contained more SCFAs, including acetate (*P* = 2.8e−08), propionate (*P* = 0.0044), butyrate (*P* = 0.0021), and isovalerate (*P* = 0.017), than the dried products. These results provide insight into SCFA-microbiota associations in Thua Nao, which may enable the development of starter cultures for SCFA-enriched Thua Nao production.

## Introduction

Thua Nao (fermented soybean) is a northern Thai food with high nutritional value and is similar to products such as natto in Japan, chongkukjang in Korea, and kinema in India^1^. Three main ingredients (i.e., soybeans, water, and natural microbes) are required to produce Thua Nao, which occurs in four major steps: soaking, boiling, fermenting, and post-fermentation. Boiling destroys non-heat-resistant germs and bacteria, thus preserving the heat-resistant bacteria. The fermentation process lasts 3 days under ambient conditions. Freshly prepared or wet Thua Nao can be steamed or roasted before consumption. Under ambient conditions, wet Thua Nao can be stored for approximately 2 days. Therefore, post-fermentation processes have been developed to prolong its shelf-life. For example, cooked Thua Nao is mashed into a circular flat disk and then sun-dried, resulting in the dried form of Thua Nao, which can be stored for several months^1^.

A previous study demonstrated the value of Thua Nao as a potential resource for bioactive and health-promoting compounds^2^. Thua Nao contains 57.22–64.78% moisture, 4.70–5.44% ash, 12.92–28.06% crude fiber, 38.94–42.06% crude protein, and 20.37–25.22% fat^3^. The potential benefits of bioactive compounds, such as phenolic compounds and antioxidant activities, are not only derived from raw materials but also released from bacteria during soybean fermentation^3^. Using culture-dependent methods, *Bacillus* species are reported to be predominant microorganisms in Thua Nao^4^. However, Thua Nao is made from a mixture of natural microflora, which cannot be easily cultured. The fermentation process with mixed microflora allows the microorganisms to help one another produce important substances related to nutrition and health, such as peptides, vitamin B, fatty acids, and short-chain fatty acids (SCFAs). Soybean digestion by the microflora creates the SCFAs, i.e., acetate, propionate, and butyrate, which serve as essential nutrients for colonic mucosal cells, thus stimulating mucosal proliferation and blood flow^5^ and controlling sugar levels^5^, metabolism^6^, and cholesterol levels^7^. Therefore, Thua Nao is considered a good source of important substances, prebiotics, and bacteria beneficial to health.

Thua Nao is produced in various regions in northern Thailand; however, data on bacterial communities in Thua Nao from different areas is still limited. Studying the bacterial communities may enable a better understanding of the roles and functional properties of fermenting bacteria in Thua Nao. The microbes used for natural fermentation originate from growing soybean. We thus hypothesized that different soybean growing regions or geographical conditions could influence the composition of the microbiota and ultimately the composition of SCFAs. Natto could be a good control compared to other fermented foods (e.g., fermented fish and pickles) because it is also a fermented soybean, but, with the addition of *Bacillus* as a starter culture. To address this, we first identify the bacterial taxa in Thua Nao from six different areas in northern Thailand using culture-independent approaches (i.e., 16S rRNA gene sequencing). Second, we analyzed the correlations between the bacterial taxa and the types and amounts of SCFAs in Thua Nao using gas–liquid chromatography. The results revealed the beneficial bacteria that may be used as starter cultures for improving Thua Nao production quality.

## Results

### Characteristics of fermented soybean (Thua Nao) in Thailand

**Figure 1:**
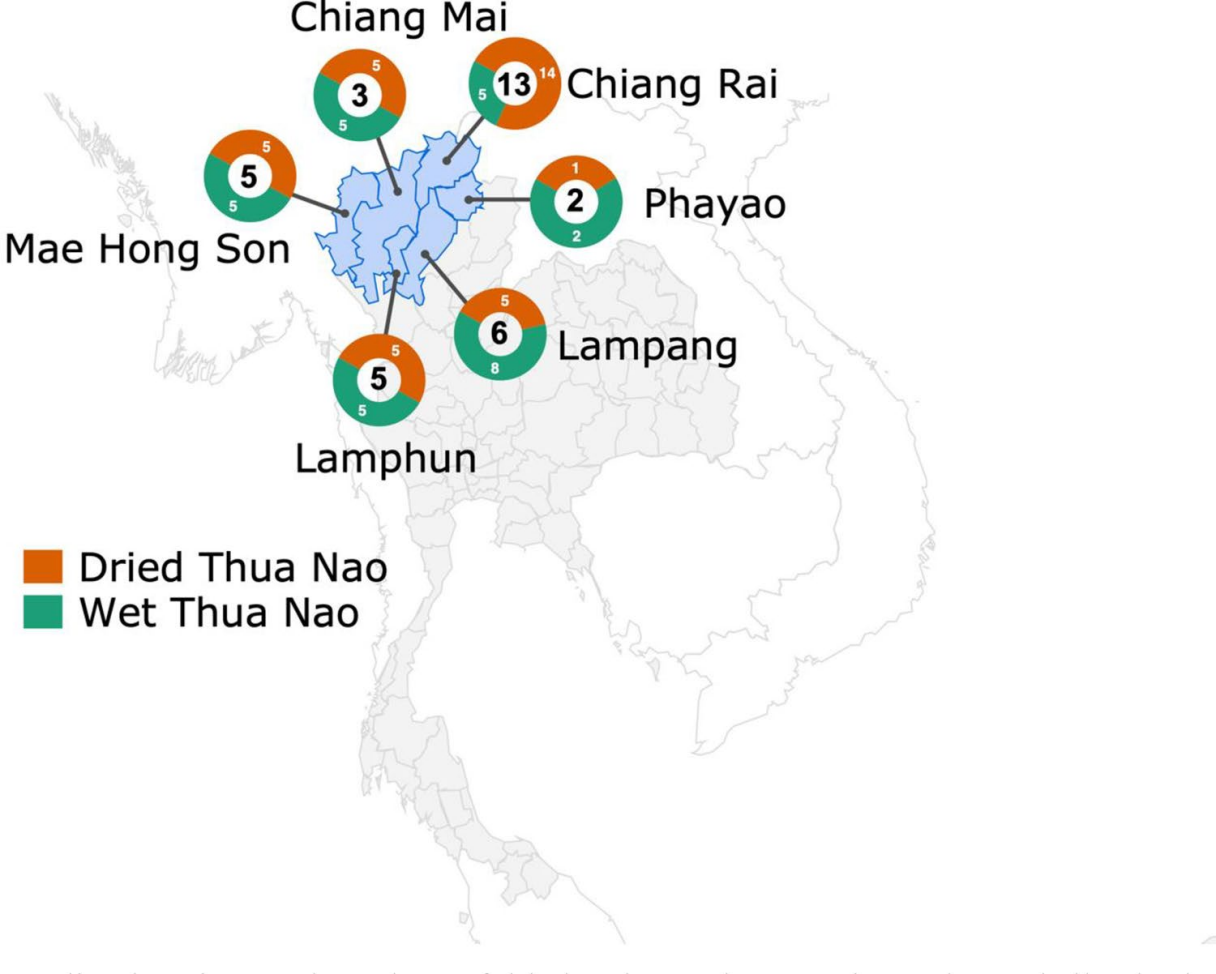
Sampling locations and numbers of dried and wet Thua Nao in northern Thailand. The number in the center of the circle represents the number of villages in each province, while the number in the orange and green colors indicates the number of dried and wet samples in each province, respectively. The map was downloaded from Google Data Studio, and additional graph and pointer were drawn using Microsoft Excel v16.66.1, Microsoft PowerPoint v16.66.1, and Inkscape v1.1.

Sixty-five Thua Nao samples, consisting of both wet and dried fermented soybeans, were collected from 37 villages in six provinces of northern Thailand. Commercial natto was also used as a non-traditional fermented soybean sample (control; n = 1), that was derived from a supermarket in Thailand. Figure 1 shows a map of Thailand with the sampling numbers in each province.

### Microbiome statistics

The median number of raw 250-bp paired-end read sequences generated by the Illumina sequencing with the 16S rRNA genes was 66,438 (range 36,740–82,116). After preprocessing, the median number of sequences was reduced to 48,645.5 (range 31,864–60,876). Sequences were then clustered into 652 features based on the amplicon sequence variant (ASV) method with most of the sequence length of 253 bp corresponding to the approximate size of the V4 region of the 16S rRNA genes. Supplementary File S1 shows the taxonomic abundance tables at the phylum and genus levels.

### Bacterial taxonomic signatures of Thua Nao

**Figure 2:**
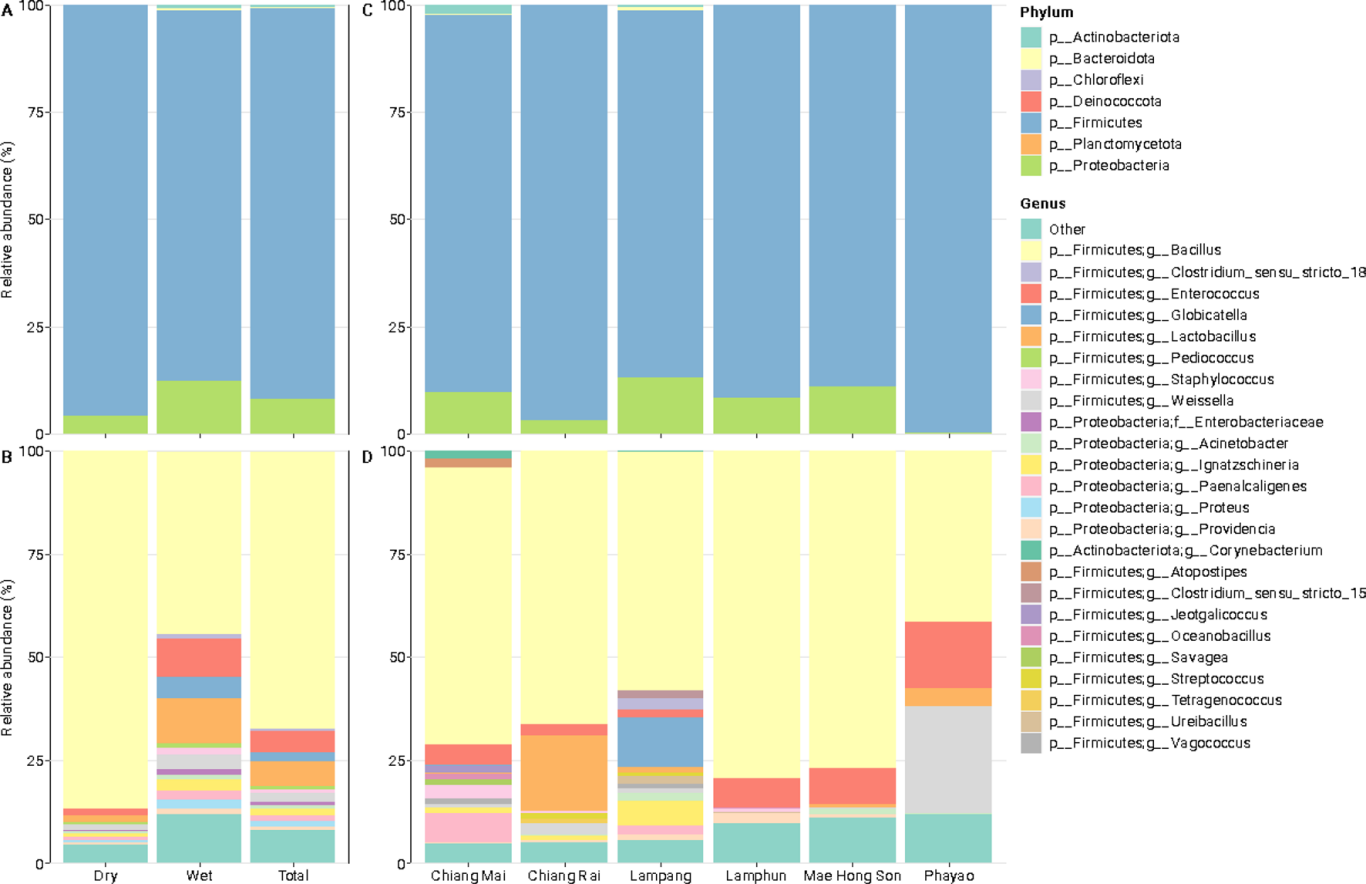
Percent relative abundances of the taxonomic compositions were compared between dried (n = 35) and wet (n = 30) fermented soybeans at the phylum (**A**) and genus (**B**) levels and among the six provinces at the phylum (**C**) and genus (**D**) levels.

Sixty-six fermented soybean samples (30 wet Thua Nao, 35 dried Thua Nao, and 1 natto) were obtained and analyzed for bacterial communities based on the diversity of 16S rRNA gene sequences. Four phyla were identified in both types of fermented soybeans. Firmicutes was the most common phylum in the dried (95.98% + 11.55%), wet (88.42% + 15.46%), and both (92.66% + 13.71%) types (Fig. 2A). The relative abundances of Proteobacteria, Actinobacteriota, and Bacteroidota were 10.26%, 0.85%, and 0.47% in the wet samples, respectively, and 3.85%, 0.08%, and 0.09% in the dried samples, respectively (Fig. 2B). Twelve genera had ≥ 1% relative abundances (Fig. 2D). *Bacillus* was the dominant genus in the dried (86.41% + 16.41%), wet (47.19% + 30.63%), and both (66.94% + 32.25%; Fig. 2D) types. Interestingly, *Lactobacillus*, *Enterococcus,* and *Globicatella* were enriched in the wet samples.

**Figure 3:**
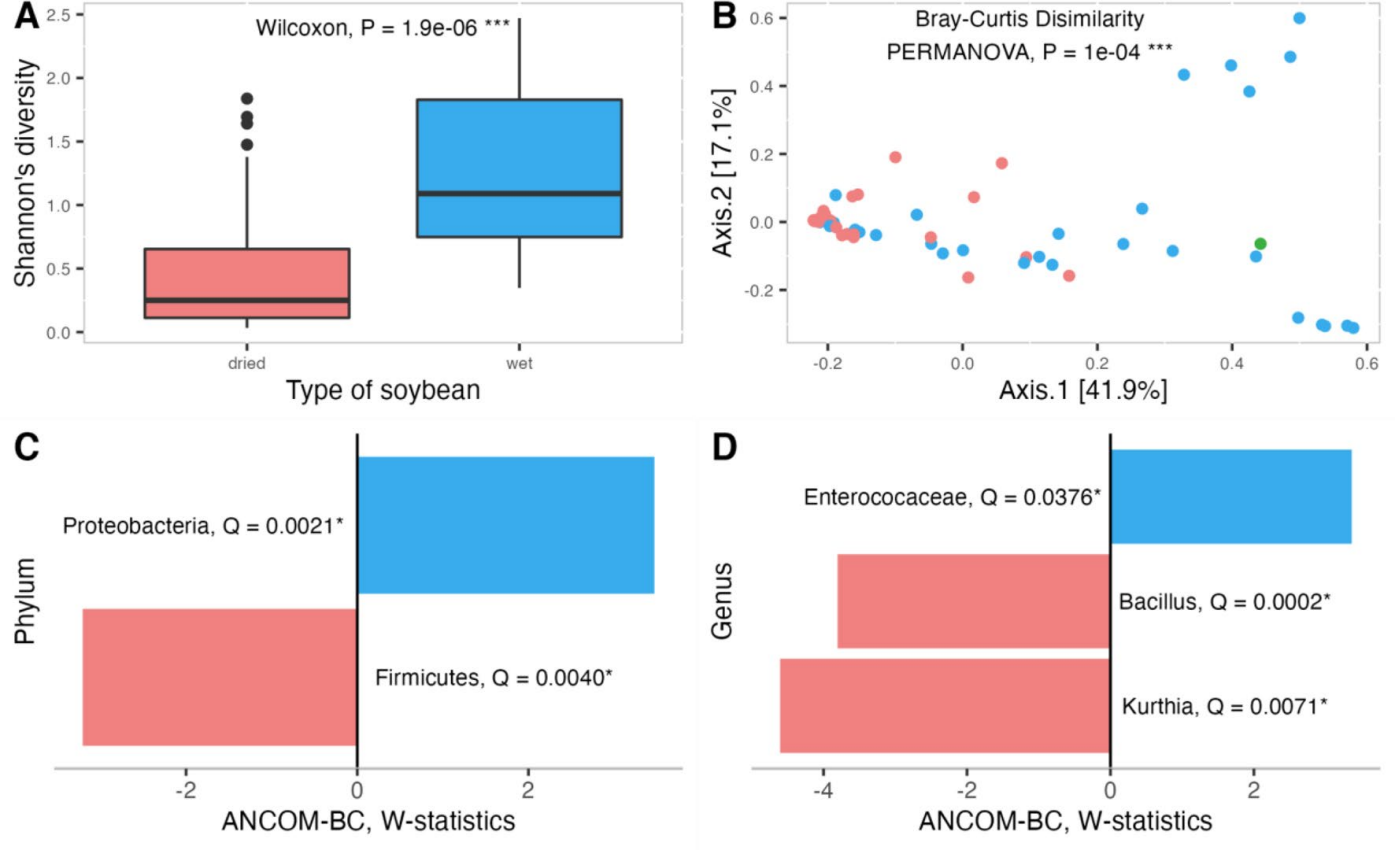
Comparison of bacterial diversities in dried (red; n = 35) and wet (blue; n = 30) fermented soybeans and natto (green; n = 1). Statistical significance tests for alpha diversity between dried and wet fermented soybeans based on Shannon’s diversity (**A**). Beta diversity among dried and wet fermented soybeans and natto based on Bray–Curtis dissimilarity and PERMANOVA (**B**). Differential abundance analysis between dried and wet fermented soybeans at the phylum (**C**) and genus (**D**) levels using ANCOM-BC methods; the Q value represents a *P*-value that has been adjusted for the False Discovery Rate (FDR).

Alpha diversities were estimated for all microbiome profiles of the fermented soybeans and compared between dried and wet fermented soybeans based on Shannon’s diversity (Fig. 3A), Simpson’s diversity (Fig. S1B), Pielou’s index (Fig. S1C), and Faith’s phylogenetic diversity (Fig. S1D) using the Wilcoxon rank-sum test. All alpha diversity metrics showed that microbial diversities of the wet fermented soybeans were significantly higher than those of the dried fermented soybeans. Beta diversities were measured between fermented soybean samples (Fig. 3 and S2). Differences between fermented soybean types were evaluated using PERMANOVA tests (Fig. 3B), which indicated that wet and dried fermented soybean and natto had different microbiota patterns (*P* = 1e−04). Differential abundant taxa between dried and wet fermented soybeans were identified with the cutoff criteria *P* < 0.05 (Fig. 3C and D).

Bacterial compositions among the fermented soybeans showed that Lampang had the most bacterial genera (14 genera) with ≥ 1% relative abundances, followed by Chiang Mai, Chiang Rai, Phayao, Mae Hong Son, and Lamphun (Fig. 2D). Interestingly, Proteobacteria had lower abundances in Chiang Rai and Phayao than in the other provinces (Fig. 2C).

**Figure 4:**
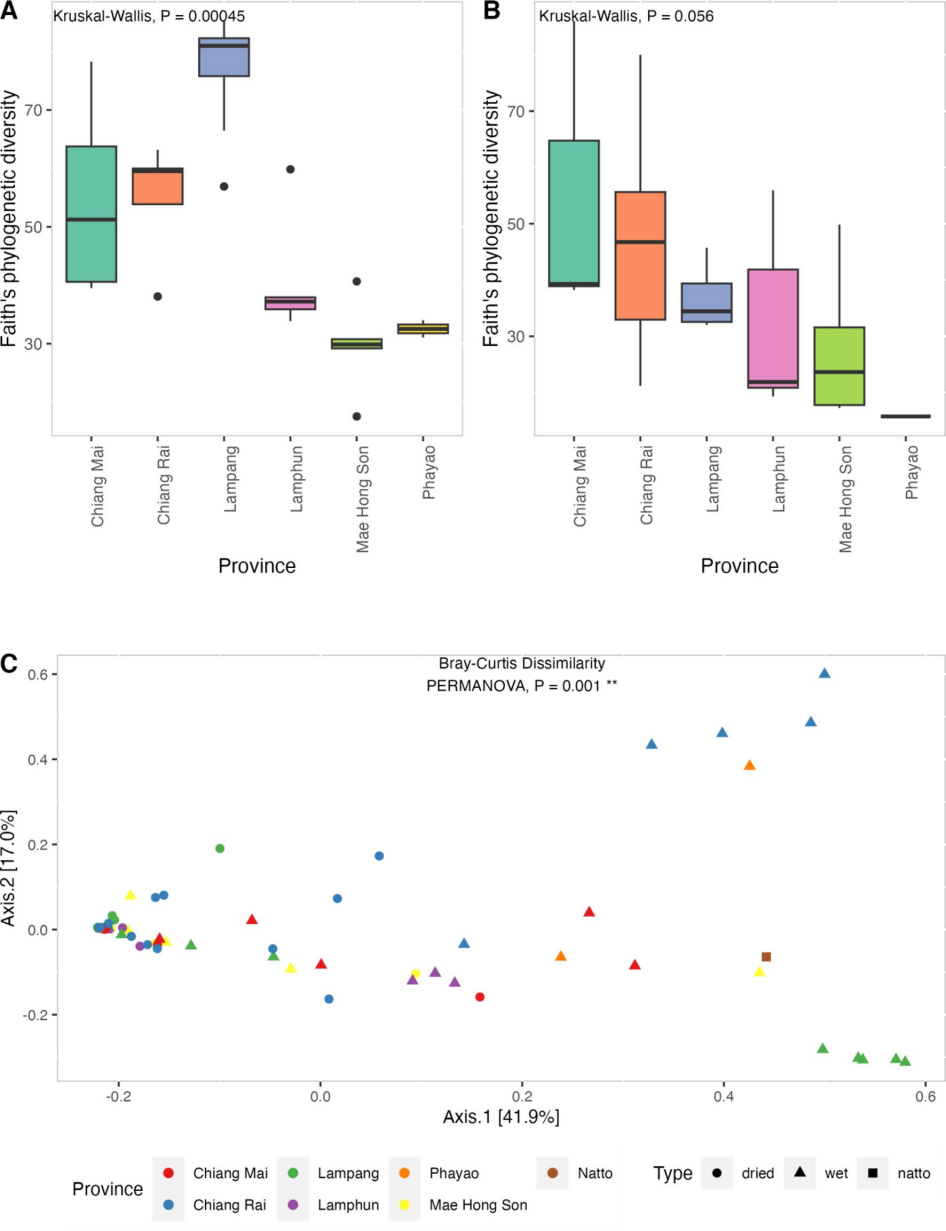
Comparison of bacterial phylogenetic diversities and dissimilarity in fermented soybeans among provinces. Statistical significance tests for alpha diversity among the provinces based on Faith’s phylogenetic diversity in wet (n = 30) (**A**) and dried (n = 35) (**B**) conditions using Kruskal–Wallis tests. Beta diversity among dried and wet fermented soybeans from six provinces and Japanese natto (n = 1) based on Bray–Curtis dissimilarity was analyzed via PERMANOVA (**C**).

Alpha diversities in terms of Shannon’s diversity, Simpson’s diversity, Pielou’s index, and Faith’s phylogenetic diversity were compared among the six provinces using Kruskal–Wallis tests (Fig. S3). The statistical results indicated that microbial diversities among the six provinces differed significantly in the wet condition based on Faith’s phylogenetic diversity (*P* = 0.0011; Fig. 4A). However, alpha diversities were not significantly different among the six provinces in the dried condition (*P* = 0.06; Fig. 4B). Alpha diversities in Chiang Rai, Chiang Mai, and Lampang were higher than those in Lamphun, Mae Hong Son, and Phayao. Differences between any two samples were measured based on Bray–Curtis dissimilarity, Jaccard distance, weighted UniFrac distance, and unweighted UniFrac distance (Fig. S4). Beta diversities based on four metrics were analyzed and visualized using principal coordinate analysis (PCoA) plots (Fig. S4). PERMANOVA indicated significant differences in the bacterial profiles from the six provinces (*P* = 0.001; Figs. 4C and S3).

### Natural SCFA levels of Thua Nao

**Figure 5:**
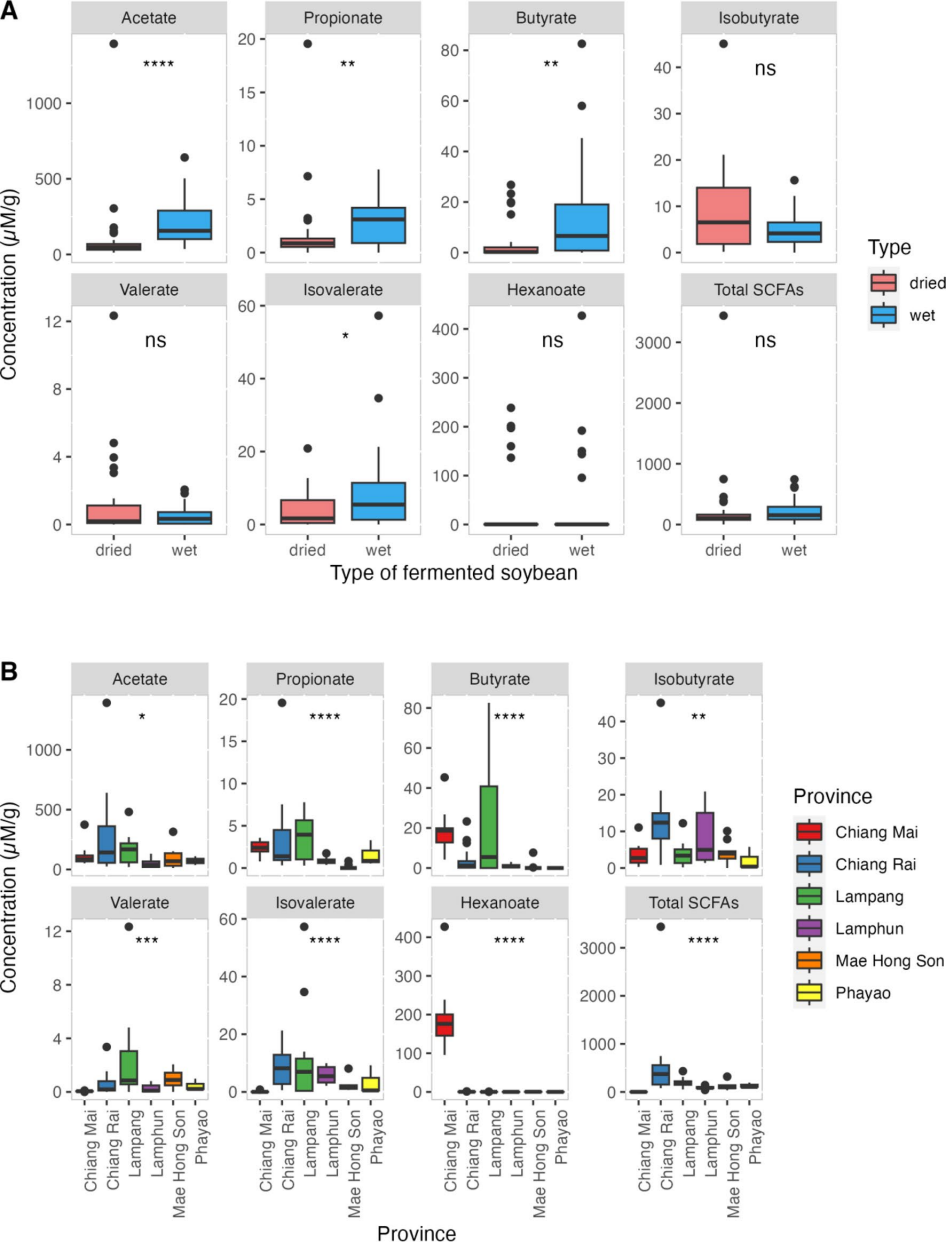
Comparison of SCFA concentrations in the fermented soybeans. Dried (n = 35) and wet (n = 30) fermented soybeans were compared using Wilcoxon’s rank-sum test (**A**) and assessed among six provinces using the Kruskal–Wallis test with a cutoff of *P* < 0.05 (**B**).

The fermented soybeans were measured for their SCFA levels. Acetate (*P* = 2.8e−08), propionate (*P* = 0.0044), butyrate (*P* = 0.0021), and isovalerate (*P* = 0.017) were greater in wet fermented soybeans (n = 30) than in dried fermented soybeans (n = 35) based on Wilcoxon rank-sum tests (Fig. 5A). The dried and wet forms of the fermented soybeans did not differ significantly for isobutyrate, valerate, hexanoate, or total SCFAs (Fig. 5A). The fermentation concentrations in the dried and wet forms of the fermented soybeans were higher for acetic acid than for the other SCFAs (Fig. 5A). Acetate and isovalerate were the most abundant in Chiang Rai and Lampang; isobutyrate and total SCFAs were dominant in Chiang Rai (Fig. 5B). Lampang and Chiang Mai contained the highest amounts of propionate and butyrate, respectively (Fig. 5B). Chiang Mai had the greatest amount of hexanoate (Fig. 5B). All six provinces had the highest concentrations of acetate fermentation in Thua Nao (Fig. 5B).

### Identification of beneficial microbes associated with SCFA production

**Figure 6:**
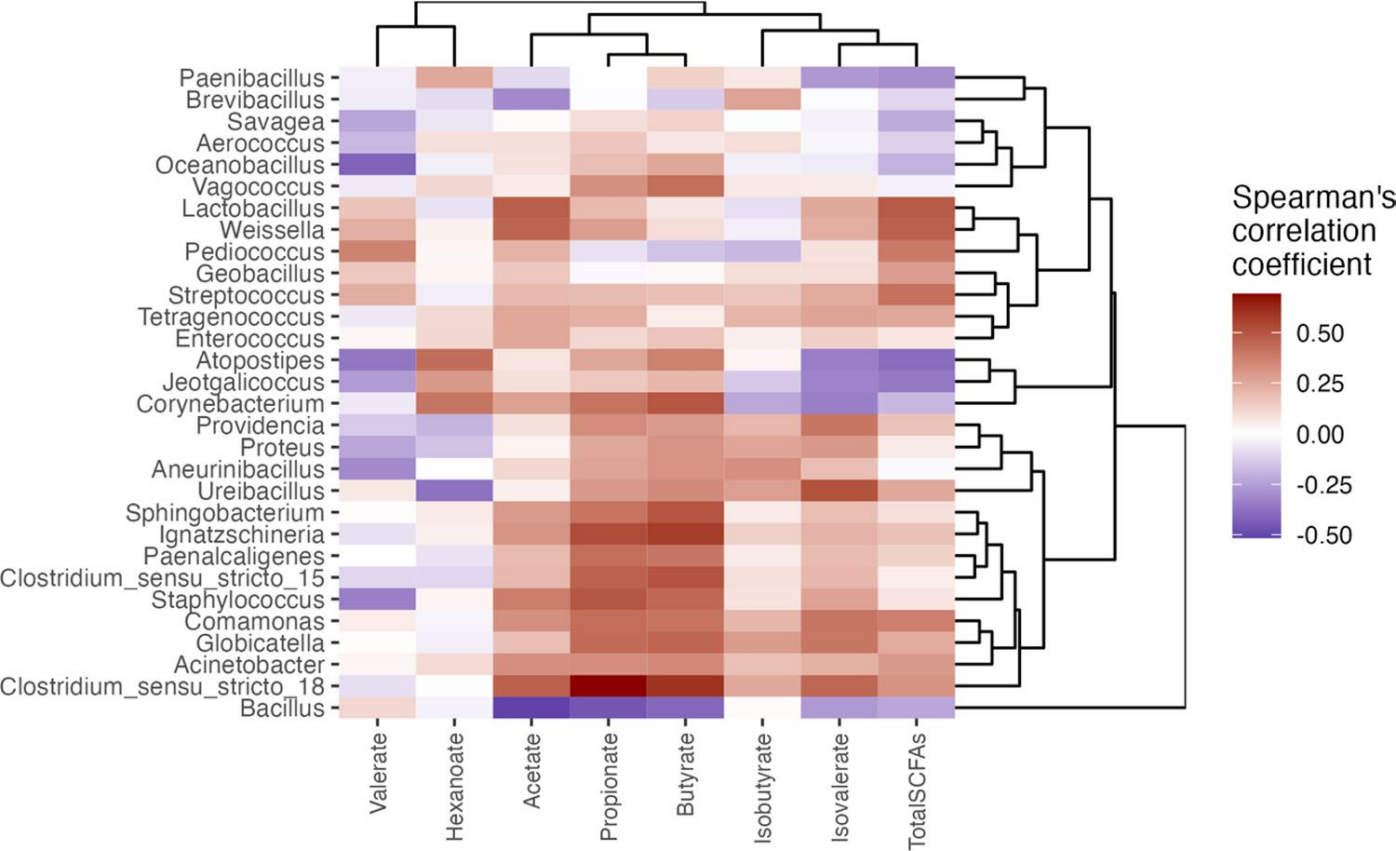
Pairwise correlations between SCFAs and dominant bacterial genera with the highest total abundances across samples of > 0.1% using Spearman’s correlation coefficient.

The correlations (R) between bacterial abundances and SCFA concentrations based on Spearman’s correlation coefficient were low and not significant (*P* > 0.05) (Fig. 6 and Table S4). The greatest R values were found between increasing *Weissella* (R = 0.45), *Lactobacillus* (R = 0.47), or *Clostridium_sensu_stricto-18* (R = 0.46) abundances and acetate concentrations (Table S4). Using a cutoff of 1% for relative abundance, *Weissella* was found in Chiang Rai, Lampang, and Phayao; *Lactobacillus* was found in Chiang Rai and Lampang, and *Clostridium_sensu_stricto-18* was found only in Lampang (Table S4). To increase propionate levels, *Globicatella* (R = 0.40), *Ignatzschineria* (R = 0.5), *Clostridium_sensu_stricto-15* (R = 0.44), *Clostridium_sensu_stricto-18* (R = 0.66), *Paenalcaligenes* (R = 0.40), or *Staphylococcus* (R = 0.46) abundances were the greatest bacterial members in fermented soybeans (Table S4). *Globicatella* and *Clostridium_sensu_stricto-15* were found in Lampang; *Ignatzschineria* was found in Lampang, Chiang Mai, and Chiang Rai; *Paenalcaligenes* was found in Lampang and Chiang Mai, and *Staphylococcus* was found in Chiang Mai and Phayao, with a ≥ 1% relative abundance (Table S3). Compared with other bacterial abundances, *Globicatella* (R = 0.44), *Ignatzschineria* (R = 0.56), *Corynebacterium* (R = 0.49), *Sphingobacterium* (R = 0.49), *Clostridium_sensu_stricto-15* (R = 0.50), *Clostridium_sensu_stricto-18* (R = 0.59), *Vagococcus* (R = 0.41), *Paenalcaligenes* (R = 0.40), *Staphylococcus* (R = 0.44), and *Comamonas* (R = 0.40) were the most impacts on increased butyrate concentrations (Table S4). In addition, increasing total SCFAs were based on increased *Weissella* (R = 0.44) and *Lactobacillus* (R = 0.46) abundances (Table S4). *Corynebacterium* was found in Chiang Mai; *Sphingobacterium* was found in Lampang; *Vagococcus* was found in Lampang and Chiang Mai, and *Comamonas* was found in Mae Hong Son, all with a ≥ 1% relative abundance (Table S3).

## Discussion

In this study, we characterized the microbiota of Thua Nao from different areas of northern Thailand and identified the beneficial bacteria potentially synthesizing SCFAs. We hypothesized that raw materials used in Thua Nao production (i.e., soybean and the natural flora of each area) would yield diverse bacterial classification results. We also assessed two types of Thua Nao, wet and dried, using 16S rRNA gene sequencing, a culture-independent method. At the phylum level, Firmicutes was the most abundant and common in both wet and dried fermented soybean products (Fig. 2A). Members of Firmicutes mainly include beneficial bacteria that play significant roles in the relationship between gut bacteria and human health. Some Firmicutes members can ferment dietary fiber from legumes to metabolite end-products, including SCFAs^8^. We further analyzed the predominant genera from Firmicutes with ≥ 1% abundances. The present results suggested that *Bacillus* was the most abundant in both wet and dried samples, which was consistent with previously reported microbial compositions^2,4,9^. As expected, *Bacillus* was also the predominant bacterial genus in the natto sample, which was the control in this work. However, we had only one sample for natto, which did not allow us to perform any statistical test between natto and Thua Nao.

Considering the differences in bacterial diversity between wet and dried Thua Nao, differential abundance analyses showed high abundances of lactic acid-producing bacteria (LAB). LAB inoculants are used to produce various animal and vegetable products and for developing flavors, aromas, and textures in fermented foods^10^. The different proportions of LAB in the wet and dried samples could be factors in the viability of LAB in preserved Thua Nao. Two LAB genera, *Lactobacillus* and *Enterococcus*, highly abundant in wet samples, have been reported to be able to hydrolyze soy proteins^11,12^. *Lactobacillus* spp. are commonly used as probiotics for human and animal supplements, and mixed cultures of *Lactobacillus* are used to ferment soybean meal^13^. Although some enterococci are considered pathogenic determinants, these bacteria also have several positive features. Many effective enterococci strains have been described as potential starters in various fermented products. The use of enterococci as starter cultures or co-cultures has increased considerably. However, the mixed natural factors of soybean substrates and other predominant bacteria (such as *Bacillus*, LAB, and other bacterial members) as well as other microbes (such as yeasts and moulds) might create unique flavors, aromas, textures, and nutritive values in wet Thua Nao.

Thua Nao is a popular fermented food in northern Thailand because it can be used as an ingredient to increase the flavor in cooked foods and can be eaten directly with glutinous rice or rice^1^. Various factors in Thua Nao manufacturing, including the types and amount of fibers in raw soybeans, the natural microbiota, environment, and fermentation duration vary among the provinces, resulting in distinct microbiome profiles and different tastes of Thua Nao. Our results indicate that Thua Nao samples from Lampang, Chiang Mai, and Chiang Rai had higher microbial diversity than those from Lamphun, Mae Hong Son, and Phayao in the wet condition (Fig. 4A).

Thua Nao contains high concentrations of non-digestible carbohydrates, which cannot be digested by endogenous enzymes in the stomach and small intestines but are a potential fermentation substrate for beneficial microbes in the gut and are considered prebiotics^14–16^. Fermentation using natural beneficial microbes in fermented food production involves hydrolyzing non-digestible carbohydrates to sugars, which are subsequently fermented to produce primarily SCFAs as well as H_2_ and CO_2_^16,17^. Several studies reported that the types and amounts of SCFAs depend greatly on bacterial digestibility and the fermentability and viscosity of fiber^16,17^. The three main SCFAs, acetic acid (C2), propionic acid (C3), and butyric acid (C4), help to release energy for gut microbial growth and physiological effects in humans^16^. SCFAs are volatile and can be produced by anaerobic fermentation of dietary fibers^6,18^. These metabolites were significantly higher in wet Thua Nao than in dried Thua Nao (Fig. 5A), possibly owing to their volatility and destroyed anaerobic enzymatic activities during the drying process^15,18,19^.

Acetic acid was the most abundant SCFA in both wet and dried Thua Nao samples. Previous studies revealed that the main acetic acid producers in the solid fermentation included anaerobic *Clostridium*, *Ignatzschineria*, LAB and aerobic acetic acid bacteria (AAB)^20–22^. In this study, *Clostridium *sensu stricto, *Ignatzschineria*, LAB (i.e., *Lactobacillus*, *Pediococcus*, *Streptococcus*, *Enterococcus*, *Globicatella*, *Vagococcus*, and *Weissella*), and AAB (i.e., *Acetobacter*) in Thua Nao might be associated with acetic acid production during fermentation. The two highest concentrations of acetic acid found in Chiang Rai and Lampang corresponded to the predominance of LAB, AAB, *Ignatzschineria,* and *Clostridium* spp.

The three major propionate formation pathways are the succinate, acrylate, and propanediol pathways^23^. *Propionibacterium* and *Clostridium*, which are anaerobes, have been explored as potential propionate producers via the succinate pathway^20,24^. Correlation analysis showed that the propionate concentrations were closely related to *Globicatella*, *Ignatzschineria*, *Clostridium *sensu stricto, *Paenalcaligenes*, and *Staphylococcus* abundances. *Clostridium*, *Ignatzschineria,* and *Paenalcaligenes* are reported to be potential propionic acid producers^25,26^. Although *Ignatzschineria* and *Paenalcaligenes* belong to the phylum Proteobacteria, implicated in human infections^27^, these genera were found in very low amounts (< 1%) in dried Thua Nao (Table S2). The highest amount of propionic acid was observed in Lampang, followed by Chiang Mai, and corresponded to the highest relative abundances of *Globicatella*, *Clostridium *sensu stricto, and *Staphylococcus*.

Correlation analysis showed that the butyrate concentrations were closely related to the abundances of *Globicatella*, *Ignatzschineria*, *Corynebacterium*, *Sphingobacterium*, *Clostridium *sensu stricto, *Vagococcus*, *Paenalcaligenes*, *Staphylococcus*, and *Comamonas*. Previous studies showed that butyrate can be generated from the fermentation of LAB such as *Globicatella*, *Vagococcus*, *Staphylococcus*; *Bacillus* spp.; *Corynebacterium*, *Comamonas*, *Sphingobacterium*, *Ignatzschineria*; and *Clostridium *sensu stricto^28–30^. The highest amount of butyric acid was observed in Chiang Mai, followed by Lampang, and corresponded to the highest relative abundances of *Corynebacterium* in Chiang Mai, *Sphingobacterium* and *Vagococcus* in Chiang Mai and Lampang, *Globicatella* and *Clostridium *sensu stricto in Lampang, and *Comamonas* in Mae Hong Son. Correlation analysis showed that the total SCFA concentrations were closely related to the relative abundances of *Weissella* and *Lactobacillus*. The highest amounts of total SCFAs observed in Chiang Rai corresponded to the highest relative abundances of *Weissella* and *Lactobacillus*.

Our results indicate that both wet and dried Thua Nao from the northern provinces of Thailand are potential sources of SCFAs. Notably, we used an amplicon-based microbiome approach to observe the bacterial communities in Thua Nao, and the results were based on genus-level bacteria. This study provides insight into SCFA-microbiota associations in Thua Nao, which could be used to develop starter culture for SCFA-enriched fermented soybean production as a supplementary food to modulate lifelong health. The effect of the microbiome in Thua Nao on SCFA fermentation end-products should be further investigated using other approaches, including shotgun metagenomics, genome sequencing, and metabolomics.

## Methods

### Sample collection

Sixty-five Thua Nao products (naturally fermented soybean samples) consisting of 30 wet and 35 dried samples were collected from 36 villages in six northern provinces of Thailand: Chiang Rai (14 dried, 5 wet), Chiang Mai (5 dried, 5 wet), Mae Hong Son (5 dried, 5 wet), Lampang (5 dried, 8 wet), Lamphun (5 dried, 5 wet), and Phayao (1 dried, 2 wet). We also used natto, a soybean from Japan fermented using a leavening agent as the control. In 27 of 36 villages, we collected both wet and dried fermented soybean samples. Moisture content was analyzed for weight determination.

### DNA extraction and 16S rRNA sequencing

Total genomic DNA was extracted from 67 samples using the DNeasy® Power Food® Microbial Kit (Qiagen, MD, USA). The DNA concentration was determined using a Nanodrop instrument (Thermo Scientific, USA). DNA integrity was monitored on 1% agarose gel. The extracted DNA samples were shipped to Genome Quebec Innovation Center (McGill University, Montreal, QC, Canada) for PCR amplification, library preparation, and sequencing. PCR was performed to produce V4 regions of the 16S rDNA gene using the conserved primers 515F (5′-GTGCCAGCMGCCGCGGTAA-3′) and 806R (5′-GGACTACHVGGGTWTCTAAT-3′). A FastStart® High Fidelity PCR System (Roche, Mannheim, Germany) was used for 26 amplification cycles. A blank sample without a DNA template was used as a negative control. The library was sequenced on an Illumina MiSeq platform per the company's protocol, and 250-bp paired-end reads were generated. A mock community (Zymo Research, USA), including 10 bacterial species, was used as a positive control.

### Determination of SCFAs in Thua Nao

SCFA analysis was performed using gas chromatography per the methods of^31,32^ with slight modifications. One gram of ground Thua Nao samples was mixed with 3 mL of internal standard solution (heptanoic acid, 5.04 µM/g sample). Samples were vortexed and centrifuged at 2,500 × g at 4 °C for 10 min, then 10 µL of 1 M phosphoric acid was added to 300 µL of the supernatant. The supernatant was filtered through a nylon filter with 0.45-µm pore size, and 0.2 μL of the filtrate was injected into a gas chromatograph equipped with a flame ionization detector (GC-FID, model 7890A; Agilent Technologies, Santa Clara, CA, USA) and capillary column (DB-FFAP, 30 m × 0.53 mm × 0.5 µm). The initial oven temperature was held at 90 °C for 1 min, then increased at 20 °C/min to 190 °C and held for 2.5 min. The injector and detector temperatures were set at 210 °C. Helium was used as a carrier gas with the gas flow and septum purge rates at 7.7 and 3.0 mL/min, respectively. A standard SCFA mixture containing acetate, propionate, butyrate, isobutyrate, valerate, isovalerate, and hexanoate as well as phenol and p-cresol was used for the calculation. The SCFA concentrations were calculated in µM/g. Quantification was performed based on the relative peak area of each SCFA standard, adjusting the quantity based on the internal standard.

### Microbiome data analysis

Sequences were 250-bp paired-end reads in the FASTQ format. Sequences were imported into QIIME2-2022.2^33^. Adapters and primers were trimmed from the front of both forward and reverse reads using q2-cutadapt. Sequences were truncated at the forward and reverse positions; paired-end reads were then merged with overlapping regions into sequences representative of V4 16S rRNA, and chimeric sequences were discarded using q2-dada2. Preprocessed sequences were clustered into features based on the amplicon sequence variant method, and the feature counts in the samples were arranged in an abundance table^34^. Features were taxonomically annotated based on SILVA v138^35^. Rarefying normalization was applied to make all samples comparable to the equal number of reads. The taxonomic abundances of all samples were calculated using q2-classify-sklearn.

### Diversity analysis

Taxonomic abundances at the genus level were used to calculate diversity metrics. Alpha diversity based on Shannon’s diversity, Simpson’s diversity, Pielou’s evenness, and Faith’s phylogenetic distance were estimated for all samples. Alpha diversity was compared between soybean types using Wilcoxon’s rank-sum tests and evaluated among provinces using Kruskal–Wallis tests. Pairwise comparisons between provinces were conducted using Wilcoxon’s rank-sum tests with multiple hypothesis corrections. Beta diversity in terms of Bray–Curtis dissimilarity, Jaccard index, UniFrac phylogenetic distance, and weighted UniFrac phylogenetic distance was calculated for all samples. PCoA was performed using Phyloseq v1.36.0^36^.

### Differential abundance analysis

ANCOM-BC v1.2.2^37–39^ was performed to identify differentially abundant microbes between wet and dried fermented soybeans with the criteria *P* < 0.05.

### Association analysis

Taxa with total relative abundances < 0.1% were filtered out from the taxonomic table. Pairwise correlations between taxonomic profiles and SCFA concentrations were calculated using Spearman’s correlation coefficient and visualized using ggplot2 v3.3.5.

### Statistical tests

Wilcoxon rank-sum tests and Kruskal–Wallis tests were conducted in R, v4.1.0. Boxplots and PCoA plots were created using ggplot2 v3.3.5.

## Supplementary Information

Supplementary Information.

## Data Availability

Sequencing data were submitted to NCBI SRA with accession numbers SRR20217885– SRR20217951 under the BioProject accession number PRJNA852866.
