# Supplementary material for: Microbiome analysis of thai traditional fermented soybeans reveals short-chain fatty acid-associated bacterial taxa
Source: Sci Rep. 2023 May 10;13:7573. doi: 10.1038/s41598-023-34818-0 (PMC10172314; doi:10.1038/s41598-023-34818-0)
Supplement: Supplementary file 1 — Supplementary Information. [file 41598_2023_34818_MOESM1_ESM.docx]

**SUPPLEMENTARY INFORMATION**

**Microbiome Analysis of Thai Traditional Fermented Soybeans Reveals Short-Chain Fatty Acid-Associated Bacterial Taxa**

Thidathip Wongsurawat^1,2,†,^*, Sawannee Sutheeworapong^3,†^, Piroon Jenjaroenpun^1,2^, Suvimol Charoensiddhi^4^, Ahmad Nuruddin Khoiri^5^, Supachai Topanurak^6^, Chantira Sutthikornchai^7^, Pornrutsami Jintaridth^8,^*

^1^Division of Medical Bioinformatics, Research Department, Faculty of Medicine Siriraj Hospital, Mahidol University, Bangkok, 10700, Thailand.

^2^Siriraj Long-read Lab (Si-LoL), Faculty of Medicine Siriraj Hospital, Bangkok, 10700, Thailand.

^3^Systems Biology and Bioinformatics Laboratory, Pilot Plant Development and Training Institute, King Mongkut’s University of Technology Thonburi, 10150, Thailand.

^4^Department of Food Science and Technology, Faculty of Agro-Industry, Kasetsart University, Bangkok, 10900, Thailand.

^5^Bioinformatics and Systems Biology Program, School of Bioresources and Technology and School of Information Technology, King Mongkut’s University of Technology Thonburi, Bangkok, 10150, Thailand.

^6^Department of Molecular Tropical Medicine and Genetics, Faculty of Tropical Medicine, Mahidol University, Bangkok, 10400, Thailand.

^7^Department of Protozoology, Faculty of Tropical Medicine, Mahidol University, Bangkok, 10400, Thailand.

^8^Department of Tropical Nutrition and Food Science, Faculty of Tropical Medicine, Mahidol University, Bangkok, 10400, Thailand.


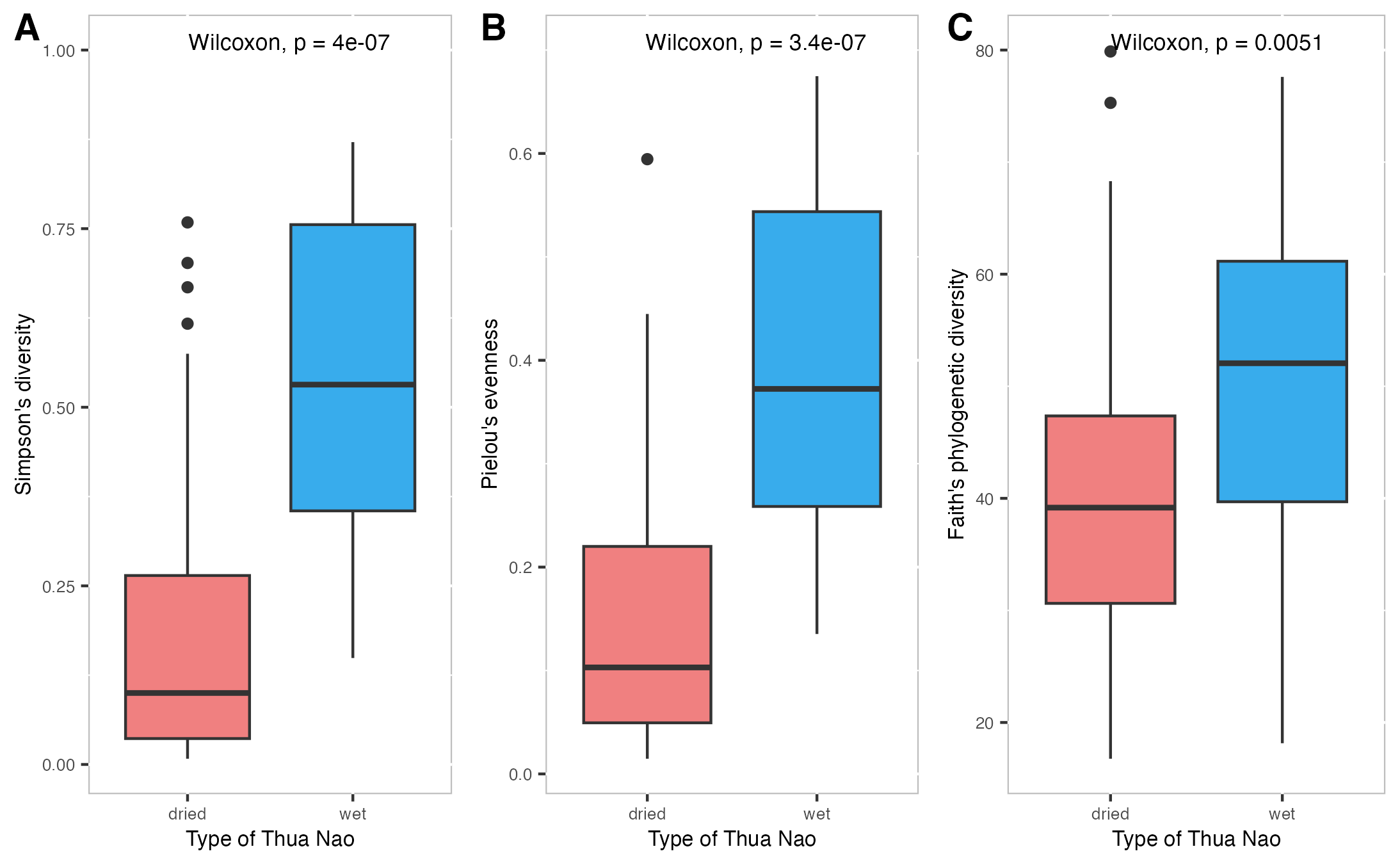


**Supplementary Figure S1** Statistical significance tests of alpha diversity between dried (red; n = 35) and wet (blue; n = 30) Thua Nao using Simpson’s diversity (A), Pielou’s evenness (B), and Faith’s phylogenetic diversity (C).

**
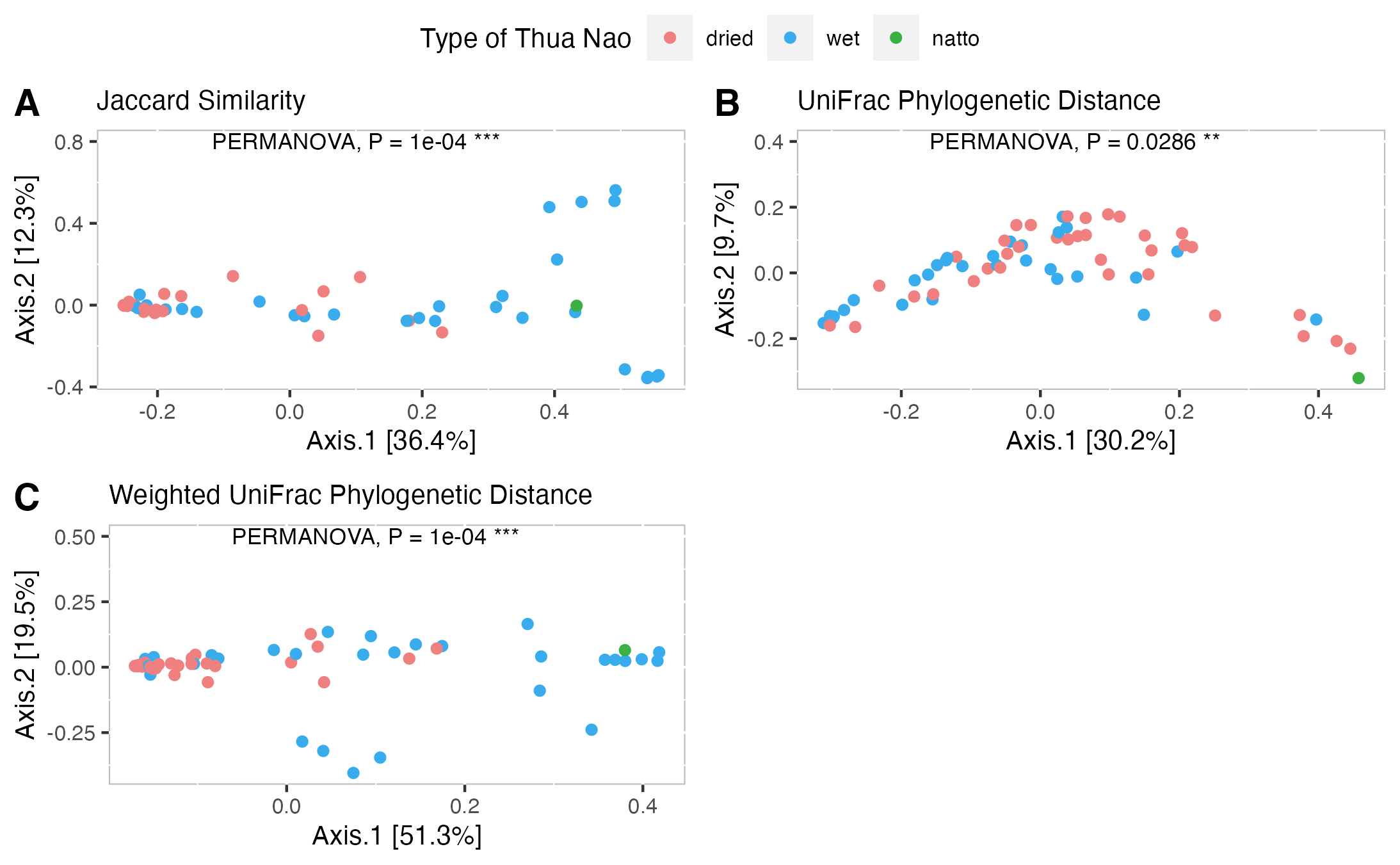
**

**Supplementary Figure S2.** Statistical significance tests of beta diversity between wet (n = 30) and dried (n = 35) Thua Nao based on Jaccard similarity (A), UniFrac phylogenetic distance (B), and Weighted UniFrac phylogenetic distance (C).


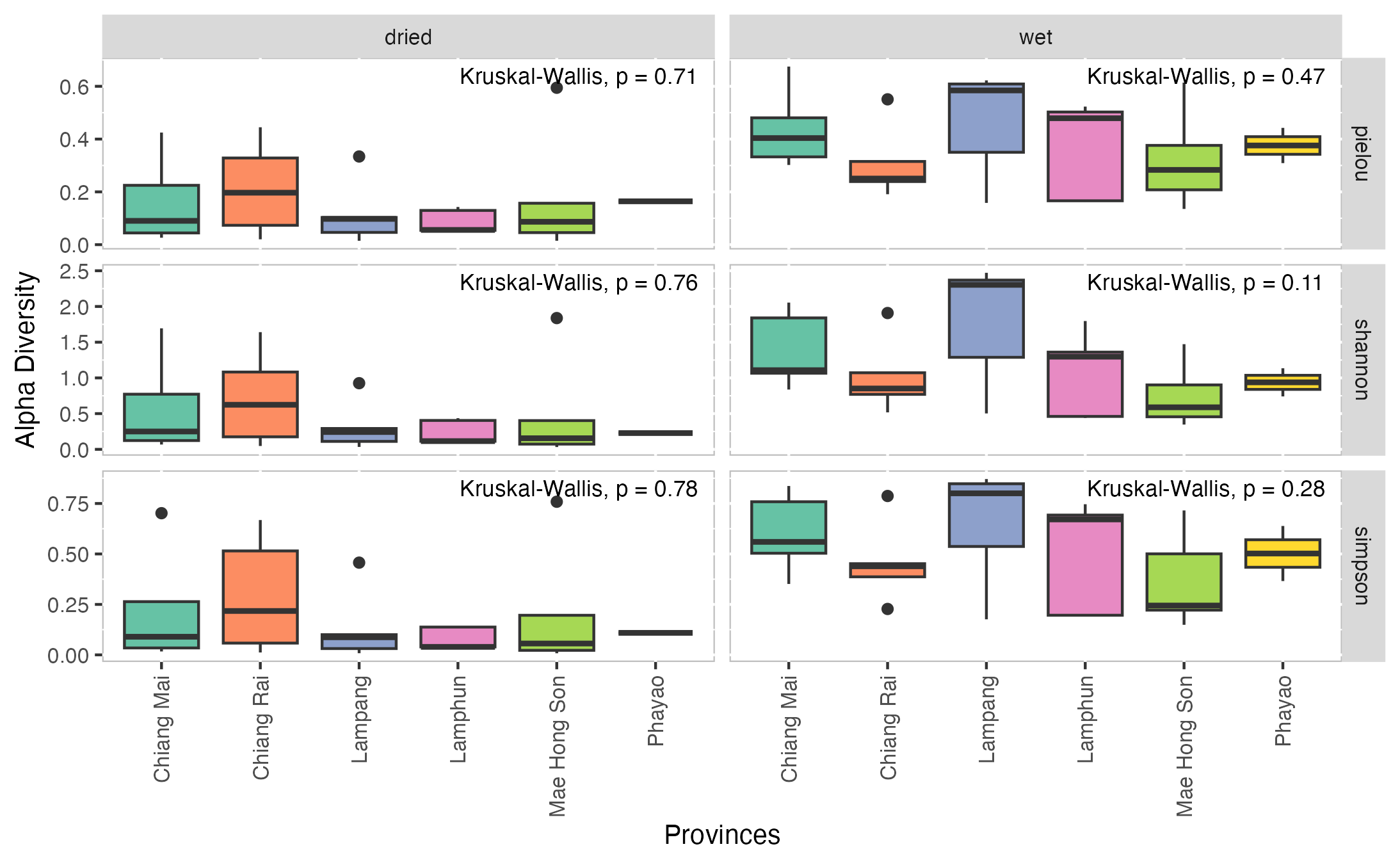


**Supplementary Figure** **S3.** Statistical significance tests of alpha diversity among provinces in the wet (n = 30) and dried (n = 35) Thua Nao using Shannon’s diversity, Simpson’s diversity, and Pielou’s evenness.


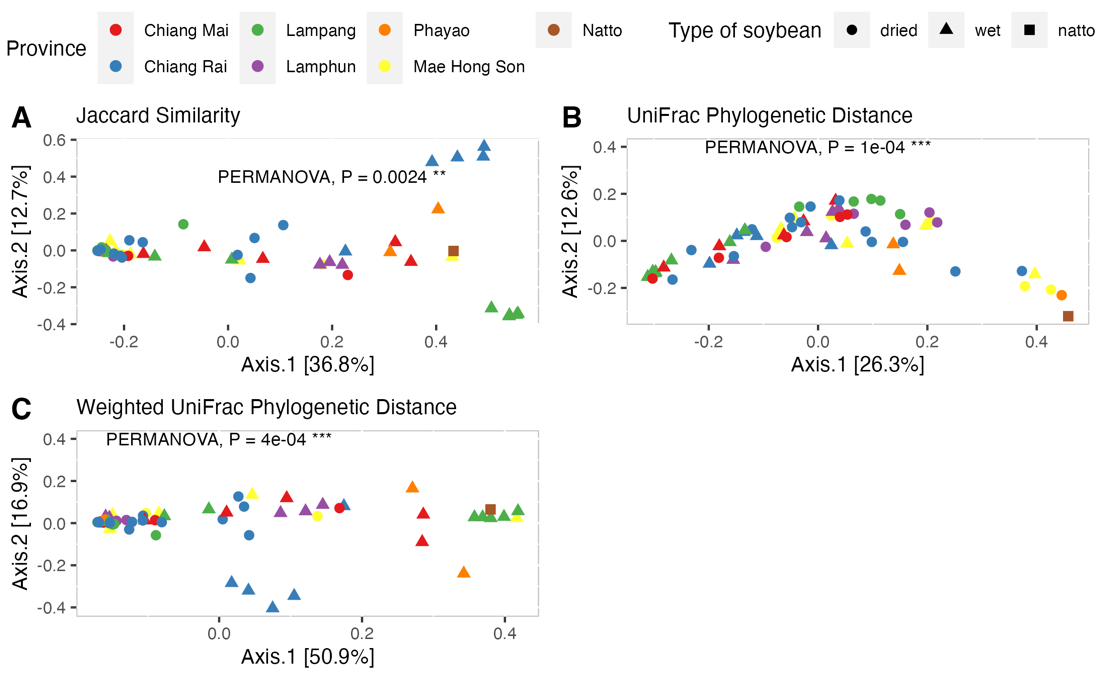


**Supplementary Figure** **S4.** Statistical significance tests of beta diversity among fermented soybeans derived from different provinces in northern Thailand including Chiang Mai (n = 10), Chiang Rai (n = 19), Lampang (n = 13), Lamphun (n = 10), Phayao (n = 3), and Mae Hong Son (n = 10) and non-traditional product (natto; n = 1) based on (A) Jaccard similarity, (B) UniFrac phylogenetic distance, and (C) Weighted UniFrac phylogenetic distance.

**Supplementary Table S1.** Differential abundant taxa between total (n=65), dried (n=35), and wet (n=30) fermented soybeans at the phylum level.

| **Phylum** | **Wet (%)** | **Dry (%)** | **Total (%)** |
| --- | --- | --- | --- |
| Actinobacteriota | 0.85+1.88 | 0.08+0.14 | 0.42+1.30 |
| Bacteroidota | 0.47+1.13 | 0.09+0.29 | 0.26+0.80 |
| Firmicutes | 88.42+15.46 | 95.98+11.55 | 92.66+13.71 |
| Proteobacteria | 10.26+14.85 | 3.85+11.42 | 6.66+13.20 |

**Supplementary Table S2.** Differential abundant taxa between total (n=65), dried (n=35), and wet (n=30) fermented soybeans at the genus level.

| **Phylum** | **Genus** | **Dry** | **Wet** | **Total** |
| --- | --- | --- | --- | --- |
| Firmicutes | *Bacillus* | 86.41+16.41 | 47.19+30.63 | 66.94+32.25 |
| Firmicutes | *Enterococcus* | 1.66+3.08 | 10.57+15.91 | 6.87+15.81 |
| Firmicutes | *Lactobacillus* | 1.59+4.13 | 10.88+26.61 | 5.69+18.20 |
| Firmicutes | *Weissella* | 1.66+4.28 | 0.89+1.85 | 2.46+10.29 |
| Firmicutes | *Globicatella* | 0.08+0.24 | 5.49+12.64 | 2.42+8.66 |
| Proteobacteria | *Ignatzschineria* | 0.8+3.16 | 2.80+6.72 | 1.63+5.05 |
| Proteobacteria | *Paenalcaligenes* | 0.91+5.29 | 2.39+7.59 | 1.51+6.32 |
| Proteobacteria | *Proteus* | 0.56+2.17 | 2.15+5.44 | 1.22+3.96 |
| Firmicutes | *Staphylococcus* | 0.2+0.65 | 1.94+6.29 | 0.95+4.21 |
| Firmicutes | *Pediococcus* | 0.60+1.40 | 1.23+4.11 | 0.87+2.89 |
| Proteobacteria | *Acinetobacter* | 0.54+1.81 | 1.88+2.04 | 0.80+1.90 |
| Proteobacteria | *Providencia* | 0.47+1.49 | 1.16+2.31 | 0.74+1.89 |

**Supplementary Table S3.** Differential abundant taxa in fermented soybeans among provinces.

| **Phylum** | **Genus** | **Chiang Rai** | **Chiang Mai** | **Mae Hong Son** | **Lampang** | **Lamphun** | **Phayao** |
| --- | --- | --- | --- | --- | --- | --- | --- |
| Firmicutes | *Bacillus* | 66.54+31.54 | 67.73+29.08 | 78.35+25.94 | 57.98+38.38 | 79.62+23.23 | 67.91+34.66 |
| Firmicutes | *Enterococcus* | 2.75+4.19 | 4.86+9.59 | 11.66+20.62 | 2.24+2.65 | 6.93+10.76 | 16.7+27.7 |
| Firmicutes | *Lactobacillus* | 18.05+31.22 | 0.28+0.87 | 0.79+1.96 | 1.26+3.42 | 0.02+0.04 | 0.41+0.71 |
| Firmicutes | *Weissella* | 2.62+5.31 | 0.75+2.33 | 0.21+0.37 | 1.14+2.15 | 0.04+0.07 | 3.1+5.36 |
| Firmicutes | *Globicatella* | 0.12+0.31 | 0.16+0.36 | 0.01+0.03 | 12.16+16.89 | 0.01+0.02 | 0.02+0.03 |
| Proteobacteria | *Ignatzschineria* | 1.01+3.63 | 1.3+3.4 | 0.0+00 | 5.92+9.26 | 0.0+0.01 | 0.0+0.0 |
| Actinobacteria | *Paenalcaligenes* | 0.00+0.01 | 7.18+15.26 | 0.0+0.0 | 2.24+3.04 | 0.00+0.00 | 0.0+0.0 |
| Proteobacteria | *Proteus* | 0.39+0.61 | 0.49+1.22 | 1.3+4.1 | 0.83+1.29 | 4.57+8.85 | 0.11+0.17 |
| Firmicutes | *Staphylococcus* | 0.71+1.79 | 3.95+10.3 | 0.00+0.01 | 0.01+0.02 | 0.7+2.0 | 1.17+2.02 |
| Firmicutes | *Pediococcus* | 0.67+1.05 | 0.0+0.0 | 1.37+3.02 | 0.7+1.91 | 0.23+0.66 | 6.8+11.78 |
| Proteobacteria | *Acinetobacter* | 0.38+0.69 | 0.15+0.23 | 1.8+3.5 | 1.99+2.51 | 0.05+0.11 | 0.11+0.19 |
| Proteobacteria | *Providencia* | 0.34+0.99 | 0.12+0.38 | 0.79+2.5 | 1.14+1.54 | 1.94+3.42 | 0.00+0.00 |
| Proteobacteria | *Comamonas* | 0.18+0.47 | 0.05+0.15 | 1.05+ 3.31 | 0.19+0.22 | 0.06+0.09 | 0.0+0.0 |
| Firmicutes | *Streptococcus* | 1.0+2.43 | 0.0+0.0 | 0.02+0.06 | 0.93+1.1 | 0.17+0.39 | 0.0+0.00 |
| Firmicutes | *Ureibacillus* | 0.04+0.13 | 0.0+0.0 | 0.0+0.0 | 1.77+2.22 | 0.05+0.08 | 0.0+0.0 |
| Firmicutes | *Vagococcus* | 0.16+0.46 | 1.28+3.41 | 0.0+0.0 | 1.09+1.52 | 0.04+0.09 | 0.0+0.0 |
| Firmicutes | *Clostridium_sensu_stricto_15* | 0.0+0.0 | 0.09+0.23 | 0.0+0.0 | 1.94+5.48 | 0.01+0.02 | 0.0+0.0 |
| Firmicutes | *Clostridium_sensu_stricto_18* | 0.06+0.11 | 0.09+0.23 | 0.0+0.0 | 2.71+3.11 | 0.0+0.0 | 0.0+0.0 |
| Bacteroides | *Sphingobacterium* | 0.1+0.32 | 0.15+0.28 | 0.01+0.02 | 1.0+1.57 | 0.06+0.18 | 0.0+0.0 |
| Firmicutes | *Atopostipes* | 0.0+0.0 | 2.03+3.34 | 0.0+0.0 | 0.0+0.0 | 0.0+0.0 | 0.0+0.0 |
| Actinobacteria | *Corynebacterium* | 0.03+0.07 | 2.03+2.81 | 0.0+0.0 | 0.18+0.22 | 0.0+0.0 | 0.01+0.01 |
| Firmicutes | *Jeotgalicoccus* | 0.0+0.0 | 1.84+5.78 | 0.0+0.0 | 0.0+0.0 | 0.0+0.0 | 0.0+0.0 |
| Firmicutes | *Brevibacillus* | 0.91+1.38 | 0.3+0.17 | 0.7+1.46 | 0.22+0.25 | 0.0+0.0 | 2.66+3.0 |

**Supplementary Table S4.** Spearman’s correlation coefficient between bacteria and SCFAs.

| **Bacteria** | **Acetate** | **Propionate** | **Isobutyrate** | **Butyrate** | **Isovalerate** | **Valerate** | **Hexanoate** | **Total SCFAs** |
| --- | --- | --- | --- | --- | --- | --- | --- | --- |
| *Weissella* | 0.45 | 0.25 | -0.06 | 0.09 | 0.22 | 0.25 | 0.04 | 0.44 |
| *Globicatella* | 0.18 | 0.4 | 0.26 | 0.44 | 0.39 | 0.02 | -0.04 | 0.23 |
| *Bacillus* | -0.51 | -0.48 | -0.02 | -0.41 | -0.29 | 0.13 | -0.03 | -0.25 |
| *Ignatzschineria* | 0.31 | 0.5 | 0.12 | 0.56 | 0.21 | -0.07 | 0.04 | 0.16 |
| *Corynebacterium* | 0.26 | 0.37 | -0.24 | 0.49 | -0.35 | -0.05 | 0.4 | -0.19 |
| *Brevibacillus* | -0.31 | -0.04 | 0.22 | -0.14 | -0.04 | -0.03 | -0.08 | -0.12 |
| *Acinetobacter* | 0.32 | 0.29 | 0.15 | 0.33 | 0.21 | 0.04 | 0.11 | 0.27 |
| *Aneurinibacillus* | 0.11 | 0.22 | 0.29 | 0.3 | 0.16 | -0.29 | 0.01 | -0.03 |
| *Lactobacillus* | 0.47 | 0.17 | -0.1 | 0.07 | 0.24 | 0.18 | -0.07 | 0.46 |
| *Sphingobacterium* | 0.29 | 0.38 | 0.04 | 0.49 | 0.18 | 0.01 | 0.06 | 0.08 |
| *Enterococcus* | 0.25 | 0.15 | 0.08 | 0.17 | 0.16 | 0 | 0.11 | 0.09 |
| *Paenibacillus* | -0.1 | -0.04 | 0.03 | 0.12 | -0.29 | -0.03 | 0.25 | -0.31 |
| *Clostridium_sensu_stricto_15* | 0.2 | 0.44 | 0.07 | 0.5 | 0.2 | -0.1 | -0.1 | 0.04 |
| *Aerococcus* | 0.09 | 0.15 | 0.09 | 0.07 | -0.03 | -0.18 | 0.09 | -0.12 |
| *Jeotgalicoccus* | 0.09 | 0.15 | -0.14 | 0.21 | -0.33 | -0.26 | 0.3 | -0.35 |
| *Ureibacillus* | 0.04 | 0.27 | 0.25 | 0.33 | 0.49 | 0.08 | -0.37 | 0.23 |
| *Streptococcus* | 0.19 | 0.17 | 0.14 | 0.17 | 0.23 | 0.24 | -0.04 | 0.39 |
| *Pediococcus* | 0.21 | -0.1 | -0.2 | -0.15 | 0.07 | 0.37 | 0.03 | 0.37 |
| *Oceanobacillus* | 0.08 | 0.17 | -0.05 | 0.26 | -0.06 | -0.41 | -0.05 | -0.21 |
| *Savagea* | 0.01 | 0.08 | -0.01 | 0.13 | -0.04 | -0.23 | -0.06 | -0.22 |
| *Clostridium_sensu_stricto_18* | 0.46 | 0.66 | 0.23 | 0.59 | 0.44 | -0.07 | 0 | 0.3 |
| *Vagococcus* | 0.05 | 0.29 | 0.05 | 0.41 | 0.05 | -0.05 | 0.11 | -0.05 |
| *Proteus* | 0.03 | 0.22 | 0.23 | 0.31 | 0.28 | -0.22 | -0.15 | 0.05 |
| *Paenalcaligenes* | 0.19 | 0.4 | 0.04 | 0.4 | 0.19 | 0.01 | -0.07 | 0.12 |
| *Geobacillus* | 0.16 | -0.02 | 0.09 | 0.02 | 0.09 | 0.16 | 0.04 | 0.28 |
| *Providencia* | 0.08 | 0.31 | 0.18 | 0.28 | 0.38 | -0.12 | -0.19 | 0.16 |
| *Staphylococcus* | 0.37 | 0.46 | 0.06 | 0.44 | 0.26 | -0.32 | 0.03 | 0.06 |
| *Comamonas* | 0.32 | 0.39 | 0.19 | 0.4 | 0.39 | 0.06 | -0.02 | 0.35 |
| *Tetragenococcus* | 0.25 | 0.22 | 0.2 | 0.05 | 0.26 | -0.05 | 0.11 | 0.25 |
| *Atopostipes* | 0.07 | 0.24 | 0.02 | 0.37 | -0.34 | -0.36 | 0.42 | -0.39 |
